# First draft genome and genomic analysis of *Neofusicoccum parvum* isolated from mango (*Mangifera indica* L.) in Paraguay

**DOI:** 10.3389/ffunb.2026.1813584

**Published:** 2026-06-05

**Authors:** Micaela Méndez, Adolfo Mateo Acuña, Mónica Morel Escobar, Mauricio Ismael González, Andrea Arrúa Alvarenga, Gilberto Benítez Rodas, Federico Da Silva, Silverio Andrés Quintana

**Affiliations:** 1 Departamento de Biotecnología, Facultad de Ciencias Exactas y Naturales, Universidad Nacional de Asunción, San Lorenzo, Paraguay; 2 Núcleo de Bioinformática y Biotecnología Integrativa, Facultad de Ciencias Exactas y Naturales, Universidad Nacional de Asunción, San Lorenzo, Paraguay; 3 Mycology Investigation and Safety Team, Centro Multidisciplinario de Investigaciones Tecnológicas, Universidad Nacional de Asunción, San Lorenzo, Paraguay; 4 Área de Gestión de Proyectos Ambientales, Centro Multidisciplinario de Investigaciones Tecnológicas, Universidad Nacional de Asunción, San Lorenzo, Paraguay; 5 Departamento de Bioquímica, Facultad de Ciencias de la Salud, Universidad Santa Clara de Asís, Caaguazú, Paraguay

**Keywords:** Botryosphaeriaceae, fungal diseases, genomic characterization, *Mangifera indica* L., *Neofusicoccum parvum*

## 1 Introduction

Mango (*Mangifera indica* L.) is one of the most economically important tropical fruit crops worldwide, with increasing relevance in Latin America for both fresh consumption and export markets (Food and Agriculture Organization, 2021; Kaur et al., 2023). Its production is significantly affected by fungal diseases, particularly post-harvest stem-end rot and fruit rot, which reduce shelf life and commercial value (Gariba et al., 2025; Guirado-Manzano et al., 2024; Lomavatu et al., 2024; Navarro et al., 2022). Members of the family Botryosphaeriaceae, including species of *Neofusicoccum*, are frequently associated with these diseases in mango-producing regions across Asia, Africa, Oceania, and the Americas (Coutinho et al., 2018; Li et al., 2021; Phillips et al., 2013; Sakalidis et al., 2011; Vecchio et al., 2025).

Among them, *Neofusicoccum parvum* is recognized as an opportunist pathogen capable of causing dieback, stem canker, and fruit rot in mango and other woody hosts. This species can persist as a latent endophyte and transition to a pathogenic phase under host stress conditions, complicating disease management and epidemiological assessments (Castaño-Tarazona et al., 2025; Hernández et al., 2023; Marques et al., 2013; Slippers and Wingfield, 2007).

Accurate identification of Botryosphaeriaceae species is challenging due to morphological similarity and the presence of cryptic species complexes. Molecular markers such as the internal transcribed spacer (ITS) region are widely used for initial taxonomic assignment, although whole-genome data provide higher resolution for species delimitation and comparative analyses (Deng et al., 2025; Sakalidis et al., 2013; Schoch et al., 2012).

Despite the cultural and economic importance of mango production in Paraguay, genomic resources for fungal pathogens associated with mango diseases in the country are currently lacking. Here, we report the first whole-genome draft sequence of *N. parvum*, obtained from a strain isolated from symptomatic mango fruits collected in the Central Department of Paraguay. The assembled and annotated genome provides a reference resource for future comparative, epidemiological, and pathogenicity-related studies of Botryosphaeriaceae in the region.

## 2 Value of data

This dataset contributes to the genomic characterization of *N. parvum* by providing, to our knowledge, the first draft assembly for this species isolated in Paraguay. While 27 N*. parvum* genome records were previously available in the NCBI database, this study increases the total to 28 and represents only the second isolate from mango worldwide, highlighting the importance of expanding host-specific genomic resources. Given that *N. parvum* is a significant causal agent of fruit rot and dieback in mango (Guirado-Manzano et al., 2024; Aiello et al., 2022), these data offer a foundational resource to investigate the genetic basis of its pathogenicity. Such information is essential for the refinement of molecular diagnostic tools and the development of effective management strategies to minimize post-harvest damage in the region.

## 3 Materials and methods

### 3.1 Fungal isolation and culture

The fungal isolate F1OG was recovered from a symptomatic mango fruit (*M. indica* L.) collected in Luque, Paraguay (25°17’58.5”S 57°30’29.7”W), on November 30, 2025. The specimen displayed characteristic symptoms of fungal infection, including dark lesions, necrotic areas, and scaly tissue. For isolation, tissue fragments were excised from the transition zone between healthy and symptomatic areas. Surface sterilization was performed by rinsing the fruits with tap water, followed by immersion in 4% sodium hypochlorite for 2 minutes and multiple rinses with sterile distilled water. The fragments were plated onto Potato Dextrose Agar (PDA) supplemented with chloramphenicol (100 mg/L) and incubated at 25 °C. Homogeneous cultures were obtained through repeated subculturing. To confirm pathogenicity, healthy mango fruits were inoculated with isolate F1OG, and the pathogen was subsequently re-isolated, successfully fulfilling Koch’s postulates (Fredricks and Relman, 1996).

### 3.2 DNA isolation and sequencing

Isolate F1OG was grown on PDA for 10 days. Mycelia were harvested by surface scraping, and total genomic DNA was extracted using the HiMedia HiPurA™ HP Fungal DNA Purification Kit, following the manufacturer’s protocol. Sequencing libraries were constructed using the TruSeq Nano DNA kit (350 bp insert size). Whole-genome sequencing (WGS) was performed on the Illumina NovaSeq X platform, generating 151 bp paired-end reads.

### 3.3 Bioinformatics and genome analysis

#### 3.3.1 Genome assembly and scaffolding

Raw read quality was assessed using FastQC v0.11.9 (Andrews, 2010). *De novo* assembly was performed with SPAdes v3.15 (Prjibelski et al., 2020). To enhance structural continuity, the resulting contigs were scaffolded using RagTag v2.1.0 (Alonge et al., 2022), employing the Complete Genome assembly of *N. parvum* DUCC19944 (NCBI Accession: GCA_020912385.1), originally isolated from a pear tree, as a reference. For the reference genome DUCC19944, which lacked prior annotation, gene prediction was performed using Augustus v3.5.0 (Stanke et al., 2006) via the Galaxy Europe platform (The Galaxy Community et al., 2024).

#### 3.3.2 Genome annotation and functional characterization

Structural annotation was carried out using the Funannotate v1.8 pipeline (Palmer and Stajich, 2020). Ab initio gene prediction was supported by GeneMark-EP (Brůna et al., 2020), integrated under a non-commercial license. Prior to annotation, the assembly was pre-processed with the funannotate clean utility to remove redundant contigs and scaffolds shorter than 1,000 bp. Evidence-based alignment was performed using the *N. parvum* NSSI1 assembly (GCA_030270365.1), isolated from *M. indica*.

Functional characterization and protein domain identification were executed using InterProScan v5.59 (Jones et al., 2014) and eggNOG-mapper v2.1.13 with the eggNOG database v5.0.2 (Huerta-Cepas et al., 2019; Cantalapiedra et al., 2021) on the Galaxy Europe platform (The Galaxy Community et al., 2024). Non-coding RNA elements, including tRNAs and rRNAs, were identified using tRNAscan-SE (Lowe and Eddy, 1997) and Barrnap v1.7 (Seemann, 2026), respectively. Secondary metabolite biosynthetic gene clusters (BGCs) were predicted using the fungal version of antiSMASH v8.0 (Blin et al., 2025). Final GFF integration and ASN.1 (.sqn) file generation were validated using the NCBI table2asn utility v1.29.324.

#### 3.2.3 Assembly evaluation and phylogenomics

Assembly quality and completeness were evaluated using QUAST v5.3 (Mikheenko et al., 2023) and BUSCO v6.0 (Tegenfeldt et al., 2025) against the ascomycota_odb12 lineage. Synteny and structural correlations between F1OG and the reference genome were visualized using D-GENIES (Cabanettes and Klopp, 2018), while a circular genomic map was constructed with Circos v0.69–8 (Krzywinski et al., 2009). The repetitive landscape was characterized using RepeatMasker v4.2.3 (Smit and Hubley, 2025) against the Dfam 3.9 database (Storer et al., 2021).

For evolutionary context, a phylogenomic tree was constructed using Buscogeny v2.1.0 (Webster and Chapman, 2026) based on single-copy orthologs. *Botryosphaeria dothidea* was selected as an outgroup. The analysis included representative genomes of *N. laricinum*, *N. kwambonambiense*, *N. cordaticola*, and *N. ribis*, alongside 27 *N. parvum* genomes available in NCBI. Phylogenetic inference was performed with 1,000 ultrafast bootstrap replicates. The final tree was visualized and styled using iTOL v6 (Letunic and Bork, 2024).

#### 3.2.4 Identification of the secretome and pathogenicity factors

To evaluate pathogenic potential, signal peptides were predicted using SignalP v6.0 (Teufel et al., 2022). To refine the secretome, TMHMM v2.0 (Krogh et al., 2001) was employed to exclude membrane-bound proteins. This analysis was conducted on the Galaxy Europe platform (The Galaxy Community et al., 2024). The secretome was further analyzed for Carbohydrate-Active Enzymes (CAZymes) using the run_dbcan v4.1.4 (Zheng et al., 2023) pipeline against the dbCAN3 database. Fungal effectors were predicted using EffectorP-fungi v3.0 (Sperschneider and Dodds, 2022). Data processing and visualization were conducted in Python 3.12.13 using pandas v2.2.2 (The pandas development team, 2026), NumPy v2.0.2 (Harris et al., 2020), Matplotlib v3.10.0 (Hunter, 2007), and Seaborn v0.13.2 (Waskom, 2021).

## 4 Dataset description

### 4.1 Genome assembly and structural annotation

**Figure 1:**
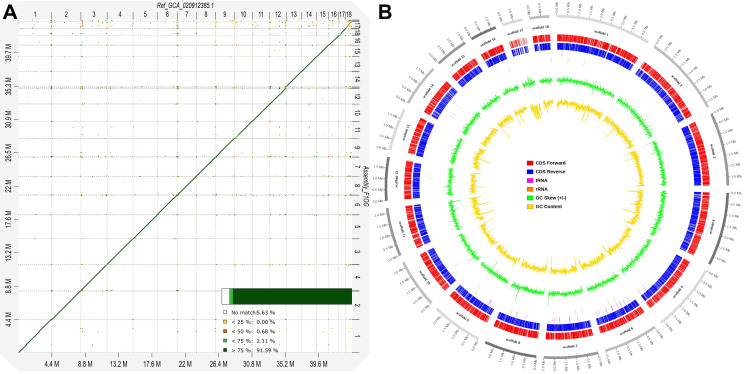
Comparative and structural genomic landscape of the *Neofusicoccum parvum* assembly. **(A)** Whole-genome dot-plot alignment generated with the D-GENIES platform. The horizontal axis represents the reference genome *N. parvum* (GCA_020912385.1), and the vertical axis represents the assembly of F1OG (Post-Scaffolding). The continuous diagonal line indicates a high degree of synteny and colinearity between the two genomes, with minor scattered dots representing repetitive elements or local rearrangements. **(B)** Circular visualization of the *N. parvum* genome architecture comprising 18 primary scaffolds. From the outermost to the innermost track, the rings represent: (i) genomic coordinates (Mb) for each scaffold; (ii) protein-coding sequences (CDS) on the forward strand (red); (iii) protein-coding sequences (CDS) on the reverse strand (blue); (iv) transfer RNA (tRNA) genes (pink); (v) ribosomal RNA (rRNA) genes (orange); (vi) GC skew (green), showing the (G-C)/(G+C) ratio; and (vii) GC content (yellow), depicting deviations from the genomic average.

The genome assembly of *N. parvum* F1OG consists of 177 scaffolds (≥1,000 bp) with a total size of 44,097,814 bp and an average sequencing depth of 30× (**Table 1**). The assembly exhibits marked syntenic conservation with the reference genome (**Figure 1A**) and is characterized by an N50 of 2,508,510 bp, an L50 of 7, and a largest scaffold of 4,396,550 bp. The GC content is 56.43%, consistent with other members of the Botryosphaeriaceae family. Genomic completeness, assessed via BUSCO, reached 99.10%, with 98.70% of genes identified as complete single copy orthologs and a total repetitive content of 2.15%.

**Table 1:** Summary of genome assembly, annotation, and quality assessment for *Neofusicoccum parvum* F1OG.

Category	Metric	Value
Assembly (Initial *de novo*)	Total assembly size (bp)	50,038,583
	Number of contigs (≥1,000 bp)	542
	Largest contig (bp)	2,028,001
	N50 (bp)	378,395
	L50	37
	GC content (%)	57.28
Assembly (Post-Scaffolding)	Total assembly size (bp)	44,097,814
	Number of scaffolds (≥1,000 bp)	177
	Largest scaffold (bp)	4,396,550
	N50 (bp)	2,508,510
	L50	7
	GC content (%)	56.43
	Sequencing coverage	30X
Genomic Annotation	Protein-coding genes (CDS)	13,054
	Total genes	13,103
	Ribosomal RNA (rRNA)	15
	Transfer RNA (tRNA)	34
Assembly Completeness (BUSCO)	Complete BUSCOs (%)	99.10%
	Complete and single-copy	98.70%
	Complete and duplicated	0.40%
	Fragmented	0.00%
	Missing	0.90%
Repetitive Content	Total repetitive content (%)	2.15%
	Retroelements (LTR/LINE)	0.56%
	Simple repeats/Low complexity	1.51%

Structural annotation identified 13,103 genes, including 13,054 protein-coding sequences (CDS), 15 ribosomal RNAs (rRNAs), and 34 transfer RNAs (tRNAs). The spatial distribution of these features is visualized in the circular genomic map (**Figure 1B**). With the inclusion of F1OG, 28 *N. parvum* assemblies are currentlyavailable in the NCBI database (**Supplementary Table 1**); however, F1OG represents only the fourth genome with detailed gene annotations, alongside strains PPO83 (isolated from *Tetrapanax papyrifer*), NSSI1 (from *M. indica*), and UCRNP2(from *Vitis vinifera*). Notably, F1OG is the first genome of this species reported from Paraguay and the second isolated from *M. indica* L. worldwide.

### 4.2 Phylogenomics and comparative analysis

**Figure 2:**
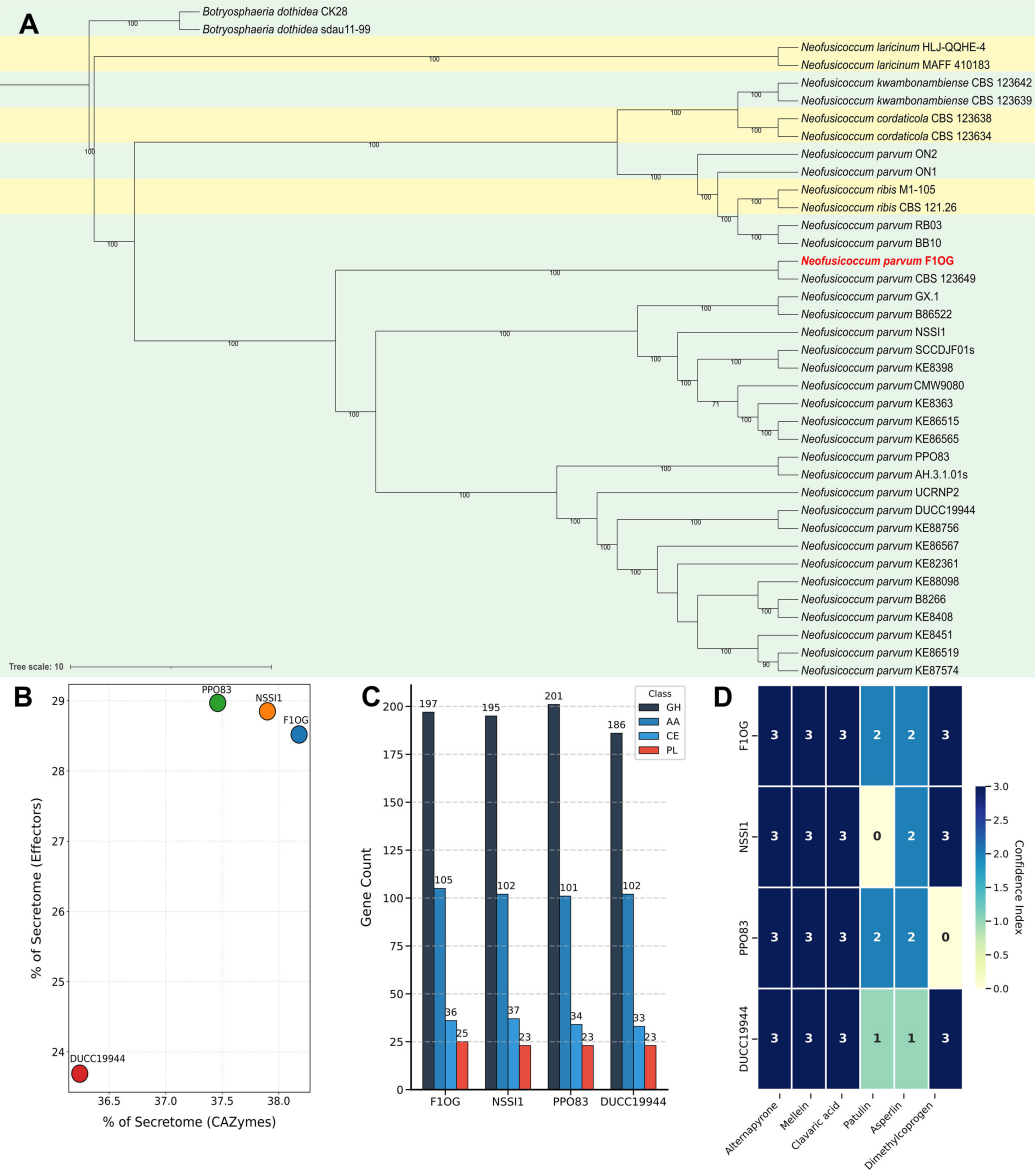
Phylogenomics, secretome distribution, and pathogenic potential of *Neofusicoccum parvum* F1OG. **(A)** Maximum-likelihood phylogenomic tree inferred from single-copy orthologs identified via Buscogeny v2.1.0. The tree was constructed with 1,000 ultrafast bootstrap replicates. *Botryosphaeria dothidea* (CK28 and sdau11-99) was used as an outgroup. Branch labels represent bootstrap support values, and the red font highlights the position of the F1OG strain within the *N. parvum* species clade. The scale bar denotes the number of substitutions per site. **(B)** Scatter plot showing the comparative investment in the high-confidence secretome. The horizontal axis represents the percentage of CAZymes relative to the total secretome, while the vertical axis represents the percentage of predicted fungal effectors. Each point represents an annotated *N. parvum* strain (F1OG, NSSI1, PPO83, and DUCC19944), highlighting the secretion profile of the mango-derived isolates. **(C)** Quantitative distribution of Carbohydrate-Active Enzyme (CAZyme) classes across the analyzed proteomes. Bars are categorized into Glycoside Hydrolases (GH), Auxiliary Activities (AA), Carbohydrate Esterases (CE), and Polysaccharide Lyases (PL), identified through the dbCAN3 HMMER-based pipeline. F1OG exhibits a robust enzymatic repertoire, particularly in GH and AA families. **(D)** Heatmap of secondary metabolite biosynthetic gene clusters (BGCs) and their associated confidence indices. Rows represent the four *N. parvum* strains, while columns list key metabolites involved in plant–pathogen interactions. The color scale and numerical values indicate the confidence index (0, absent; 1, low; 2, medium; 3, high), based on cluster orthology, completeness, and sequence similarity thresholds. Specifically, cluster similarity was categorized as high (≥ 75%), medium (50–75%), and low (15–50%), while similarities below 15% were not considered significant and are excluded from the overview. A value of 0 was assigned to clusters not present in a given strain. The heatmap highlights the presence of the patulin cluster in strain F1OG.

Phylogenomic inference based on single copy orthologs confirmed that F1OG clusters strictly within the *N. parvum* core clade, clearly resolving it from the *N. parvum*/*N. ribis* species complex (**Figure 2A**). Although the reference genome used for RagTag scaffolding (DUCC19944) provides a robust structural framework due to its hybrid assembly (Illumina and PacBio), the study isolate exhibited a greater phylogenetic affinity to *N. parvum* CBS 123649.

### 4.3 Secretome and secondary metabolism

The secretome profiles of F1OG and related isolates reveal a conserved investment in virulence factors (**Figure 2B**). F1OG maintains a robust repertoire of CAZymes, including 197 Glycoside Hydrolases (GH), 105 Auxiliary Activities (AA), 36 Carbohydrate Esterases (CE), and 25 Pectate Lyases (PL) (**Figure 2C**). This enzymatic diversity underscores its potent capacity for plant cell wall degradation.

Analysis of secondary metabolism identified conserved biosynthetic gene clusters (BGCs) for mellein and clavaric acid across all compared strains (**Figure 2D**). Interestingly, F1OG harbors a patulin BGC identified with medium confidence, based on a cluster similarity threshold of 50–75%. This feature is absent in the other mango-derived isolate (NSSI1) and shows only low confidence in DUCC19944, but is shared with the virulent strain PPO83. These results suggest that *N. parvum* isolates exhibit subtle variations in their secondary metabolic potential. Overall, this dataset provides a genomic and functional blueprint of *N. parvum* F1OG, demonstrating substantial consistency with established reference lineage.

## 5 Limitations

While the *N. parvum* F1OG assembly provides a comprehensive genomic resource with substantial gene-level completeness, it is important to note that the use of Illumina short-read technology and reference-guided scaffolding is optimized for sequence accuracy and synteny assessment. This approach, while robust for functional genomics and comparative studies, may not fully capture the complete landscape of repetitive regions or large-scale structural variations that could be unique to this regional isolate. Consequently, this draft genome serves as a reliable baseline for current epidemiological and pathogenic research in the region, while providing a foundation for future refinements using complementary long-read sequencing technologies.

## Data Availability

The original contributions presented in the study are publicly available. This data can be found here: NCBI BioProject PRJNA1420210, Genome Assembly accession GCA_056114585.1, BioSample ID SAMN55211723, and Sequence Read Archive (SRA) SRR37154756.
